# Cardio-respiratory-gated proton pencil beam scanning for cardiac ablation: workflow and end-to-end validation

**DOI:** 10.3389/fonc.2026.1923998

**Published:** 2026-10-23

**Authors:** Nicolas Depauw, Felicitas Bijari, Ethan Cascio, Evan Driscoll, Adriano Garonna, Jagdip Kang, Helen Deng, Hyeongseok Kim, Marina Macdonald-Soccorso, Willian Nett, Marc Normandin, Matthieu Pellissier-Tanon, Rebecca Woodcock, Koushiar Yaghoubian, Georges El Fakhri, Robert Levine, H.Immo Lehmann, Yen-Lin Chen, Kyung-Wook Jee

**Affiliations:** 1 Mass General Brigham, Harvard Medical School, Boston, MA, United States; 2 Yale University, New Haven, CT, United States; 3 EBAMed SA, Geneva, Switzerland; 4 Massachusetts Institute of Technology, Cambridge, MA, United States; 5 Pyramid Technical Consultants, Waltham, MA, United States; 6 University of Utah, Salt Lake City, UT, United States

**Keywords:** gating, motion management, proton therapy, stereotactic arrhythmia radio ablation (STAR), ventricular tachycardia (VT)

## Abstract

**Background:**

Stereotactic Arrhythmia Radiotherapy (STAR) offers a non-invasive alternative for ventricular tachycardia ablation, yet motion management remains a critical challenge. While respiratory gating is established, cardiac gating requires a dedicated workflow and gating device.

**Methods:**

This study establishes and validates an end-to-end workflow for cardiorespiratory gated proton STAR in six healthy swine. Key innovations include a novel pencil-beam scanning (PBS) controller enabling mid-spot beam pausing and resumption, and the integration of CardioKit, an ultrasound-based image-guidance system for real-time soft-tissue tracking. The workflow was evaluated by comparing single-gated (respiratory only) and dual-gated (cardiorespiratory) delivery strategies, assessing system latency, dosimetric accuracy on dynamic phantoms, and motion tracking fidelity against 4D-CT benchmarks.

**Results:**

The upgraded PBS system demonstrated a gate response latency of <550 µs, effectively mitigating interplay effects in dynamic phantom measurements through gated beam deliveries. CardioKit provided respiratory motion traces highly correlated with 4D-CT data (Pearson r > 0.97) and responded to arrhythmic events within 5 ms. *In vivo*, dual-gating restricted delivery to the diastolic window (70–100% R–R), which resulted in a reduction in the Internal Target Volume (ITV) expansion by a mean of 45% compared to single-gating (19% vs. 64%), thereby substantially sparing healthy myocardium. Although dual-gating increased total beam-on time, the mid-spot interrupt capability ensured robust dose accumulation without treatment aborts.

**Conclusion:**

The workflow for imaging, treatment planning, and cardiorespiratory-gated beam delivery was viable for treating all six subjects. The PBS system responded to gating commands < 550 µs and CardioKit responded to irregularities in heartbeat in <5 ms. Cardiorespiratory gating enabled an average 45% reduction in target expansion due to cardiac motion. Comparison of CardioKit respiratory motion traces with respiratory 4D-CT yielded differences of 1.1-1.4 mm in total motion amplitude and 0.97-0.99 Pearson correlation factor. These findings are valuable for establishing a workflow for cardiorespiratory-gated STAR treatment with CardioKit.

## 1 Introduction

While radiotherapy of cardiac lesions has been historically limited, given the low prevalence of cardiac-originated malignancies (1, 2), non-invasive stereotactic arrhythmia radiotherapy (STAR) is emerging as a radiation-based alternative to catheter-based ablation of ventricular arrhythmia targets. The first clinical evidence has shown that this new treatment modality is safe and can effectively reduce the Ventricular Tachycardia (VT) burden (3, 4). Most patients to date have been treated with photon STAR, but treatment is also feasible with protons (5) which offers the potential to increase STAR safety (6) thanks to its distal fall-off and high dose-conformality.

Cardiac motion is complex due to its multiple components (heartbeat-related and respiration-related, among others), patient specificity, and significant amplitude (7–10). Gating the external beam delivery based on real-time cardiorespiratory motion information allows, in principle, to increase the sparing of healthy tissues in proximity to the target from unnecessary radiation exposure. No system is yet available for standard clinical use, however.

With the advantages of its non-ionizing nature and the ability to visualize and track internal anatomy rather than external surrogates (11, 12), ultrasound imaging has been used for image-guided radiotherapy, mostly in the context of prostate and liver cancer treatments (13–16). The Clarity and Clarity Autoscan (Elekta AB, Sweden) have been the most successful ultrasound image-guided solutions in external beam radiotherapy (17–19). More recent experimental systems have relied on robotic approaches and tracking of a specific anatomical structure or region of interest (20, 21). The main limitations to the application of such systems to STAR are the low image acquisition rate and long processing time, the strong user-dependent accuracy and the stringent requirement for high-quality images for manual contouring (13–16).

Specifically for STAR, a novel ultrasound-based image-guidance system for cardiorespiratory gating - called CardioKit (EBAMed SA, Geneva, Switzerland) - was developed (22, 23).

To enable gated delivery at the Massachusetts General Hospital (MGH) Francis H. Burr Proton Therapy Center, an upgrade was made to the dose delivery system. Pencil beam scanning (PBS) and double-scattering are two distinct methods used in proton therapy to shape and deliver the radiation beam, with the former magnetically steering a narrow beam to “paint” the tumor layer by layer for high precision, while the latter physically spreads a wider beam to cover the target area. Respiratory gating had previously been implemented at MGH in double-scattering mode (24). However, no gating capability existed for the PBS mode at the institution. The development described here extends the existing gating system to PBS and further introduces fast cardiac gating, enabling the beam to be interrupted and resumed mid-stream within an individual dose spot. This is a critical capability given the sub-second timescales of cardiac motion (25). In addition, the workflow needs to be adapted, considering the integration of all necessary tools and methods.

This work describes the novel workflow to image, plan, and deliver proton therapy to a left ventricle target and presents the results from an end-to-end validation of the upgraded PBS and CardioKit in a large animal study involving six healthy swine. Other aspects related to this work have been reported elsewhere (26–30).

## 2 Materials and methods

### 2.1 Gated ablation workflow

The large-animal study protocol was reviewed and approved by the Massachusetts General Hospital Institutional Animal Care and Use Committee (IACUC). Six healthy male Yucatan pigs were used in this study.

#### 2.1.1 Simulation

Anesthetized and mechanically ventilated subjects were immobilized in the supine feet-first orientation on a Vac-Lok cushion (CQ Medical, Avondale, PA, USA). The ventilator was set with tidal volume of 5–10 mL/kg (dependent on the subject weight), inspiration:expiration ratio of 1:2, respiratory rate of 12–25 breaths per minute depending on the subject and the CO_2_ levels at each time point). Electrocardiographic (ECG) electrodes were placed on the subject’s abdomen for ultrasound imaging and cardiac-binned Computed Tomography (CT). Ultrasound imaging was performed to determine an adequate probe position for visualizing the left ventricle clearly and for treatment planning to avoid interception of treatment beams with the probe area. The CardioKit ultrasound probe was positioned using guidance from the Graphical User Interface (GUI) and the skin-adhesive probe holder was applied for hands-free imaging (22). Parasternal echocardiographic imaging was performed in free breathing, preceded by an initial short expiratory breath-hold. The probe was then replaced with a 3D plastic replica (“dummy probe” – see **Figure 1**), maintaining the same orientation in the probe holder. This dummy probe appears as a radio-opaque object in the simulation CT and can thus be easily contoured.

**Figure 1:**
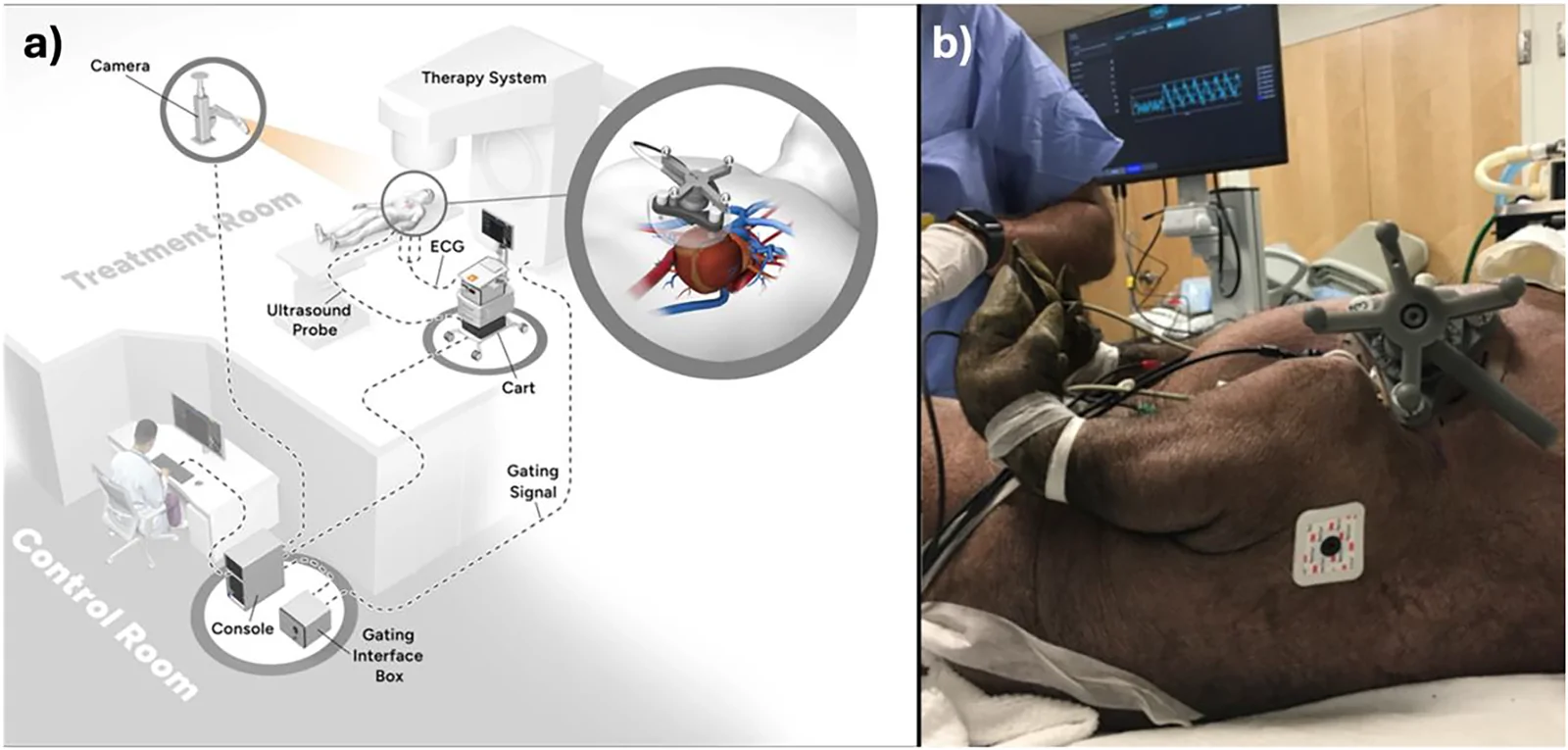
**(a)** Schematic of CardioKit components installed in a radiotherapy room (adapted from 23), similarly to how it was used in the animal study. **(b)** Picture of the CardioKit cart (background) and animal with the CardioKit dummy probe (foreground). The dummy probe is a radio-opaque 3D replica of the probe geometry and is used to visualize the probe position in the simulation CT.

Simulation CT imaging was performed on a GE Discovery MI Positron Emission Tomography (PET)/CT scanner (GE Healthcare, Chicago, IL, USA) with a flat couch. Prior to the experiment, a stoichiometric calibration of the CT (31) was performed using a Gammex 467 tissue characterization phantom (Gammex, Middleton, WI, USA), characterizing CT’s Hounsfield Unit values to proton relative stopping powers. Additionally, a calibration of the CardioKit tracking camera was performed using a phantom with internal radio-opaque ball bearings and external infra-red reflecting spheres. This is required to visualize motion in the CardioKit GUI in the room coordinate system (32).

Breath-hold CTs were acquired at end-exhalation: a pre-contrast and a contrast-enhanced ECG-gated dynamic cardiac CT were obtained and retrospectively reconstructed into 10% R–R-interval phases to generate a 4D CT dataset. Skin tattoos and radio-opaque markers for alignment were positioned according to the CT internal lasers.

#### 2.1.2 Treatment planning

Cardiac target volumes (CardTV) were defined at the quiescent cardiac phase (80% R-R) of the contrast CT by consensus of a radiation oncologist and cardiologist, encompassing the left ventricle free wall (LVFW) and papillary muscle. CardTV was defined as the intersection of the LVFW with a sphere of 3.5 cm diameter (Cases 1–2) or 2.5 cm diameter (Cases 3–6). Internal target volumes (ITV) were derived by expanding the CardTV to encompass intra-fractional cardiac motion using the deformable registration tool of MIM v.7.4.2 (MIM Software Inc, USA). For dual-gated subjects, the expansion accounted for motion within the diastolic gating window (70%–100% of the R–R interval), whereas for single-gated subjects, a larger expansion encompassing all cardiac phases was applied. Planning target volumes (PTV) were generated by adding an isotropic 5 mm margin to the ITV to account for setup uncertainty and residual respiratory motion. The 5 mm margin accounts for subject positioning uncertainties based on bi-planar kV imaging. The residual respiratory motion is set to 2 mm by configuring this gating window threshold with respect to the end-exhale ultrasound baseline: as a total 2 mm window for one-dimensional displacement in each spatial coordinate (for the first two test animals) and for a three-dimensional vector norm (for all subsequent animals). This PTV margin can be further reduced, for example, as low as 2–3 mm (33–35) if respiratory-gated CT imaging is available for subject positioning. All organs at risk (OARs) were initially contoured on the 80% phase CT and expanded by the corresponding cardiac motion envelope. The dummy probe was also contoured and considered to avoid the treatment beam paths intercepting the probe (22). Contours were transferred to the average pre-contrast CT for radiotherapy planning and dose optimization.

All plans delivered 35 GyRBE (RBE = 1.1) in a single fraction to the LVFW target using two PBS fields with 12 cm apertures (a beam-shaping device utilized to enhance the conformality of the delivered dose) and energies ranging from 100 to 150 MeV. Target coverage was optimized while enforcing a maximum dose limit to the Left Anterior Descending coronary artery (LAD), mimicking the typical clinical protocol in radiotherapy - for all but the first case of the protocol for which no OAR limit was considered. The treatment beams avoided intersection with the probe and holder.

Respiratory gating alone remains susceptible to residual interplay effects from motion within the gating window (36). For this reason, the energy layer-by-layer repainting technique – where each spot was divided into four split deliveries – was utilized for the three cases in the single-gated arm to compensate for the unrestricted cardiac motion, ensuring appropriate treatment delivery robustness (37).

#### 2.1.3 Subject setup

Subject anesthesia, intubation, venous access, and a pre-treatment echocardiogram were completed before transporting to the radiation therapy facility. Before subject arrival, a calibration of the CardioKit tracking camera was performed using the same phantom used at simulation imaging. The subject was then immobilized in the same Vac-Lok cushion of the simulation imaging. The ECG electrodes were placed, and the CardioKit probe was positioned using the probe holder markings noted at simulation imaging (22). A first subject alignment was made using the in-room lasers and markings on the skin and immobilization device. The image-guided subject setup was aided by the two orthogonal, gantry-mounted 2-dimensional x-ray system.

#### 2.1.4 Treatment delivery

For irradiation, an IBA (Ion Beam Applications, Louvain-La-Neuve, Belgium) proton system with a 235 MeV isochronous cyclotron at the MGH Francis H. Burr Proton Therapy Center (Boston, MA, USA) was used. A gantry beamline enabled optimal beam configurations. The interface between the CardioKit and the scanning controller was tested prior to the first subject irradiation. In particular, the latencies and gating signal constraints (minimal and maximal durations of gate ON/OFF commands) of the scanning controller were measured and used to configure the CardioKit software (for latency compensation).

### 2.2 Pencil beam scanning proton beam system

#### 2.2.1 Novel scanning controller

PBS is controlled by a Pyramid Technical Consultants (Waltham, MA, USA) scanning controller, which manages the beam independently of the upstream IBA cyclotron and energy selection system. The scanning controller governs all spot-by-spot delivery decisions, turning the beam on and off between each spot position. The mid-spot interrupt-and-resume capability represented the key advancement over a prior prototype, which observed the gate signal only at spot boundaries and could not pause delivery within a spot. The production design was specifically required for cardiac gating because the diastolic gating window (70%–100% of the R–R interval) is shorter than the delivery time of individual dose spots at clinically relevant dose rates (i.e. larger than 300 ms for a 25 Gy treatment plan). The novel beam-enable control logic with gating signals, as well as the various safety checks implemented, are detailed in the **Supplementary Sections 1**, **Supplementary Sections 2**. The interface to the scanning controller is through a 5V Transistor-Transistor Logic (TTL) signal.

#### 2.2.2 Latency characterization

System latency—defined as the elapsed time between a gate-on/off command and the rise/cessation of beam current at the cyclotron output—was measured prior to subject irradiation using overlaid plots of the CardioKit output gate signal and the cyclotron integrated current (IC Cyclo) monitor ion chamber. Simultaneous measurements of the two signals were performed using a fast Personal Computer-based oscilloscope (Pico Technology Ltd., Cambridgeshire, UK), which provides multi-channel time-domain acquisition up to 100 MHz analog bandwidth and a maximum real-time sampling rate of 1 GS/s, an acquisition speed well suited to capturing and time-stamping the sub-millisecond signal transitions involved in the gating latency measurement. Both the delayed onset and offset of beam current following the gating signal were characterized, as these site-specific parameters must be configured in the CardioKit software.

#### 2.2.3 Functional test of gated scanning

Functional testing of the novel scanning controller was performed using a two-dimensional Lynx scintillation detector (IBA Dosimetry GmbH, Schwarzenbruck, Germany) mounted on a CIRS dynamic thorax phantom (Dynamic SBRT Thorax and Platform Combination, VA, USA) with motion patterns mimicking respiratory (a motion period of 5 sec) or cardiac (1 sec) conditions for 1 cm and 2 cm peak-to-peak amplitudes. Deliveries of a uniform 40 × 50 mm² field with approximately a 30% gating duty cycle were compared against deliveries without gating to assess the motion mitigation of the interplay effect.

### 2.3 Cardiorespiratory gating device

#### 2.3.1 Description

The CardioKit is a novel, investigational ultrasound-based image-guidance system for target cardiorespiratory motion monitoring and gating, specifically developed for STAR. The system combines hands-free transthoracic echocardiographic and electrocardiographic data streams from the patient to provide real-time soft tissue images and ECG before and during treatment. The use of optical tracking of the ultrasound probe on the patient thorax allows the determination of the left ventricle target’s three-dimensional motion in the same coordinates as the simulation imaging and treatment delivery systems (32), using calibration methods derived from ultrasound-based navigation systems (38–42). The device and its components are shown in **Figure 1**. The device’s image and signal processing pipeline has been presented in previous work (23). In short, each ultrasound image is binned in terms of its cardiac phase and registered to the baseline ultrasound image of the corresponding cardiac phase to compute the respiratory displacement in the three spatial coordinates (or as the norm of the vector). This displacement therefore does not include the motion linked to heartbeat contraction. Heart drift during the course of treatment will appear as a non-zero minimal respiratory displacement. CardioKit is specifically designed for single-fraction large dose treatments, to ensure safe and durable operation. CardioKit can be used to provide respiratory-only gating, cardiac-only gating (not used in this study) or combined cardiorespiratory gating signal. In this study, the (single) gating signal provided to the proton therapy system is a 5 V TTL signal.

#### 2.3.2 Comparison with 4-D CT imaging

Additional imaging (not required by the workflow) was performed for two cases with the goal of using this data for preliminary validation purposes. A respiratory-gated 4D-CT was acquired with the subject in supine position, using the Real-time Position Management System RPM (Varian Medical Systems Inc, USA) with 10 bins (from 0% to 90%). Prior to this, a 30 s long sequence was acquired with CardioKit. The left ventricle was manually contoured on the 80% respiratory phase of the 4D-CT, and the contour was propagated using deformable image registration in MIM. The propagated contours were visually checked for accuracy. The coordinate of the centroid of the left ventricle was exported for analysis. The position and respiratory phase trace of the RPM marker block and the CardioKit motion trace database were exported for analysis. For the 4D-CT and CardioKit data, the three spatial coordinates were converted into the norm of the 3D vector. The RPM provides one-dimensional position coordinate of its marker block. For all the datasets, for each breathing cycle, the motion amplitude was offset to set the minimal position (i.e. end expiration) at zero. To allow temporal comparison to the other two datasets, the timestamps of the CardioKit respiratory motion trace were converted into respiratory phase using a peak detection algorithm. The comparison of the motion traces was performed in terms of total breathing motion excursion and Pearson correlation coefficient. The animals were mechanically ventilated, thus ensuring that the 4D-CT, RPM, and CardioKit data can be compared, although not acquired synchronously.

#### 2.3.3 Management of irregular heart rhythm

CardioKit automatically detects and pauses operation in the occurrence of heartbeat rhythms below 40 beats per minute (BPM) or above 120 BPM, beat-to-beat R-R interval duration changes larger than ± 5% or premature (atrial/ventricular) contractions. The ECG signal is digitized, and the signal is processed electronically to determine the peak of the R-wave. Normal operation automatically resumes after the heartbeat has stabilized. The system was tested using the ProSim 3 Vital Signs Simulator (Fluke Biomedical Inc., USA) and the Advanced Modular Patient Simulator AMPS-1 (Datrend Systems Inc., USA) with the following relevant simulator functions: atrial fibrillation (coarse), premature atrial contraction, frequent multifocal premature ventricular contractions (PVC), bigeminy, trigeminy, pair of PVCs, 5 PVCs, 11 PVCs and VT. The full list is tested for all ECG lead configurations (Lead I, Lead II and Lead III). The management of irregular heart rhythms (similarly to the other safety mechanisms for patient motion, irregular breathing and device operation malfunction among others) is handled internally and the interruption of gating is transmitted to the therapy machine via the standard gating interface.

### 2.4 Summary of datasets and analysis

A summary of the main datasets acquired, and their analysis is provided in **Table 1**.

**Table 1:** Summary of main analysis and associated datasets.

Analysis performed	Associated dataset
Latency characterization	Beam-current signal Gating signal
Dosimetric test of scanning controller	Scintillation detector 2D data
Comparison of respiratory motion curves	Respiratory 4D-CT RPM CardioKit database
Management of irregular heart rhythm	CardioKit gating signal ECG traces
Treatment planning	End-exhale pre-contrast CT ECG-gated 4D CT
Beam delivery performance	Procedure reports PBS delivery log data

## 3 Results

### 3.1 Validation of gated pencil beam scanning

#### 3.1.1 Beam-on and off characteristic performance

As shown in **Figure 2a**, measurements of the gate-on/off signals and the beam current from the cyclotron confirmed proper gated spot splitting and low system latency.

**Figure 2:**
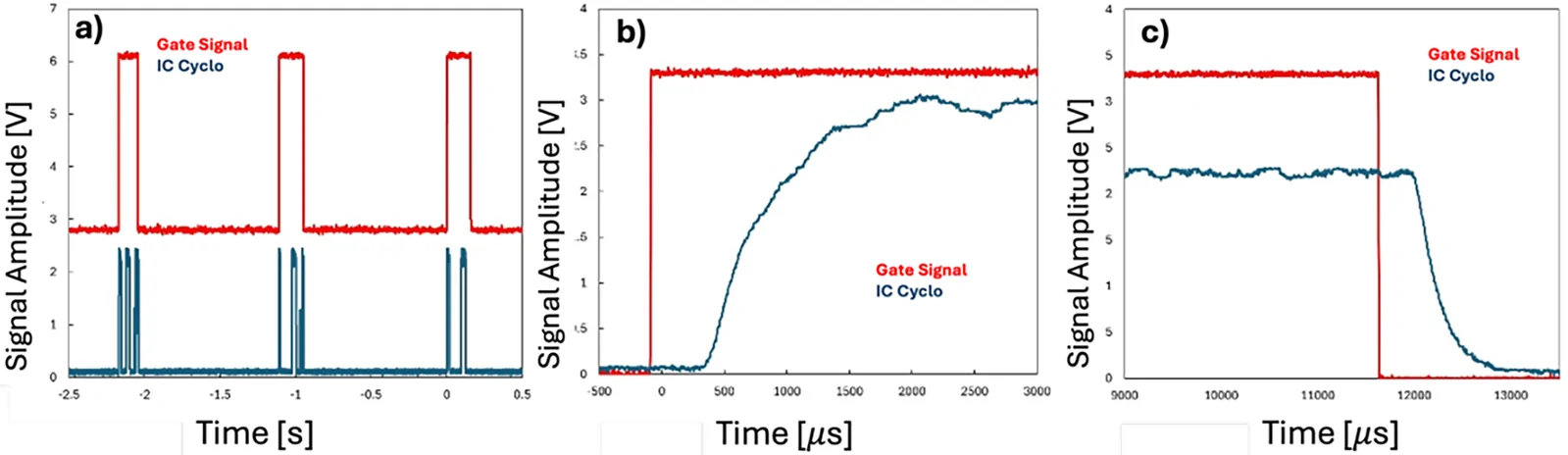
Beam current at the cyclotron exit in blue and gating signal in red for three gating pulses **(a)**, at the beginning of a gating pulse **(b)** and at the end of a gating pulse **(c)**.

A detailed analysis of spot splitting for the six subject irradiations is provided in Sec. 3.3.3. At maximum beam current, the ramp-up/down latency was measured to be less than 550 µs. The full beam current ramp-up and ramp-down times were approximately 2 ms and 1 ms, respectively.

#### 3.1.2 Dosimetric validation of gating

Measurements with a motion phantom revealed that asynchronous motion between the detector and the beam spot during a uniform 40 × 50 mm² field scan greatly degraded the planned dose uniformity. While the extent of dose inhomogeneity varied with the temporal and spatial patterns of the two moving objects and their phase differences, these motion effects were largely mitigated by gated scanning delivery. For example, a 1-second periodic, 1cm peak-to-peak phantom motion degraded dose uniformity by approximately 21.6% (shown in **Figure 3**), but this was largely restored to 6.5% by gating with an approximate 30% duty cycle (**Figure 3**).

**Figure 3:**
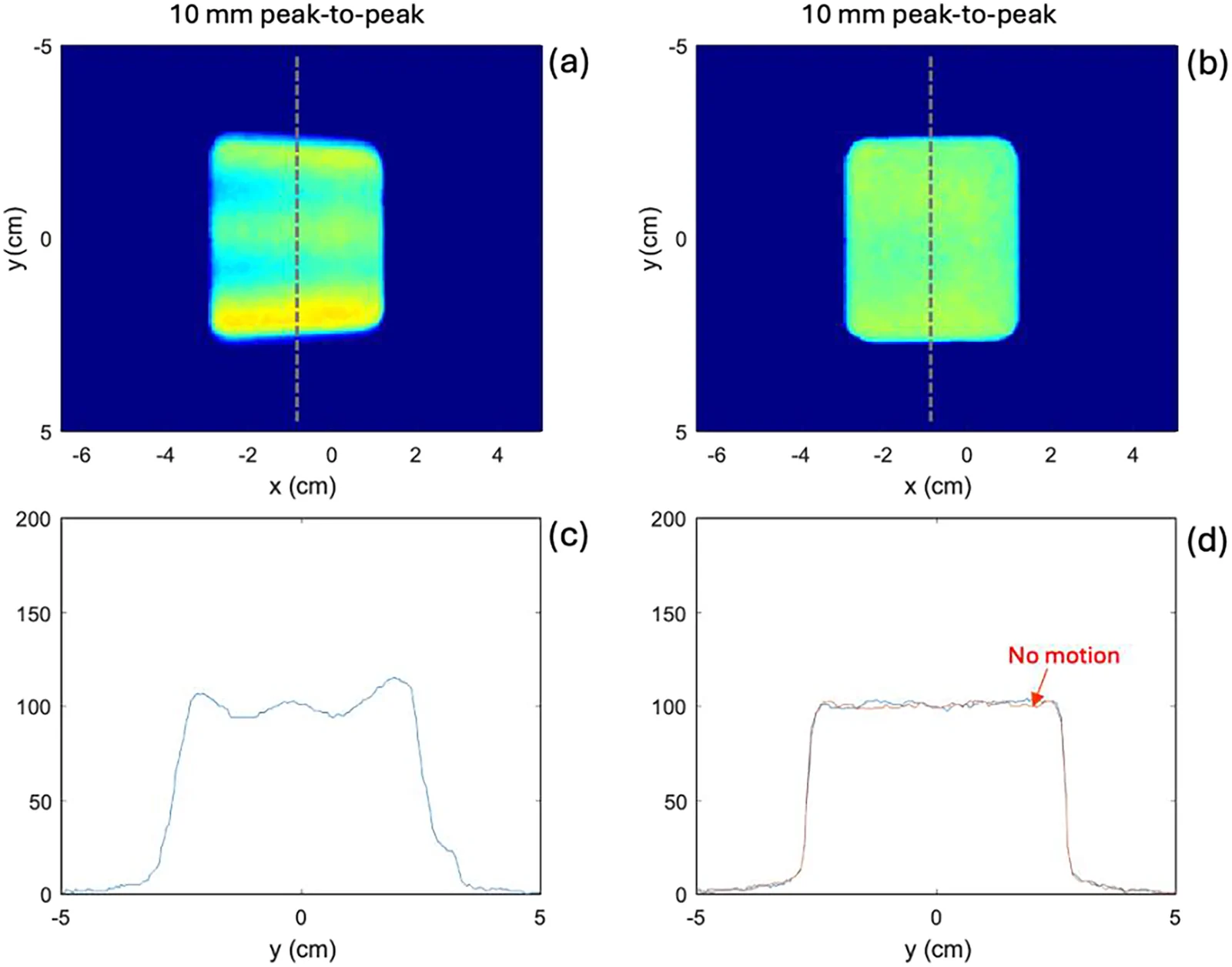
Upper figures show the 2D doses measured by the detector on a moving phantom exhibiting a 10 mm peak-to-peak motion for ungated **(a)** and gated **(b)** irradiations. Lower figures **(c, d)** show the corresponding Y-directional profiles of the deposited dose. The red profile in **(d)** corresponds to the dose profile measured without the motion of the detector.

### 3.2 Characterization of cardiorespiratory gating device

#### 3.2.1 Ultrasound-based respiratory motion accuracy

For case 3, the recorded respiratory motion amplitude (maximum excursion) was 3.4 mm (3D vector norm, left ventricle) from the respiratory 4D-CT (N = 1), 4.5 mm ± 0.3 mm (3D vector norm, left ventricle) from CardioKit (N = 7) and 1.8 mm ± 0.1 mm (1D, lower abdomen) from RPM (N = 31). For case 6, the recorded respiratory motion amplitude was 2.4 mm (3D, left ventricle) from the respiratory 4D-CT (N = 1), 3.8 mm ± 0.3 mm (3D, left ventricle) from CardioKit (N = 6) and 3.2 mm ± 0.1 mm (1D, lower abdomen) from RPM (N = 23). The Pearson correlation between the 4D-CT and the CardioKit respiratory motion traces was 0.99 and 0.97 for case 3 and case 6 respectively. The Pearson correlation between the 4D-CT and the RPM respiratory motion traces was 0.95 and 0.97 for case 3 and case 6 respectively. The comparison of the three respiratory motion traces for both subjects is graphically shown in **Figure 4**.

**Figure 4:**
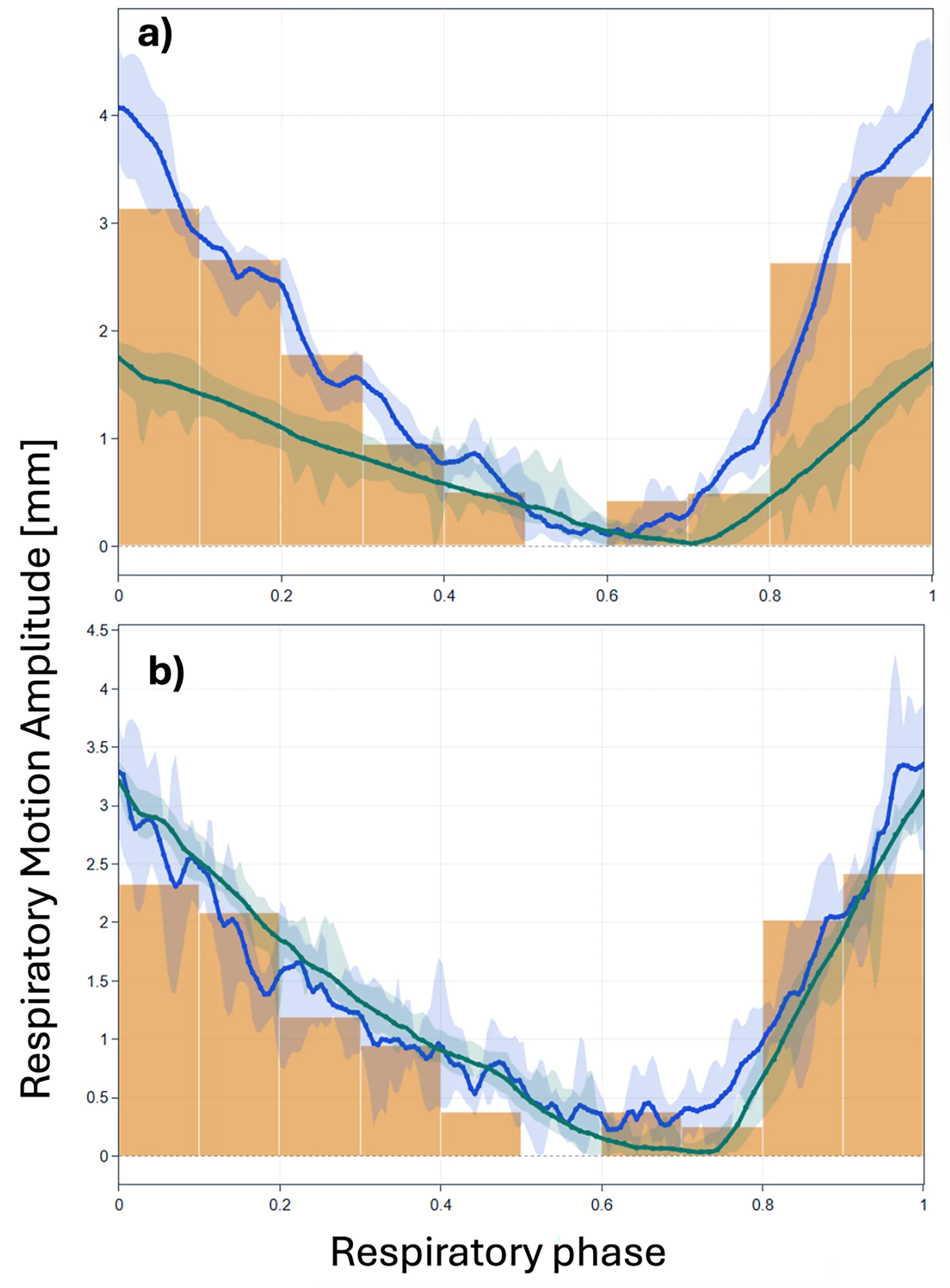
Results of the comparison of the respiratory motion curves for case 3 **(a)** and case 6 **(b)** from the 4D-CT in orange bars (3D, left ventricle), CardioKit in blue (3D, left ventricle) and RPM in green (1D, surrogate). The 4D-CT has one dataset per respiratory phase bin. The CardioKit and RPM data are shown as average motion curve (line) and minimum and maximum envelopes (shaded area).

#### 3.2.2 Management of heartbeat irregularities

Simulated beat-to-beat heartbeat duration variations larger than 5% triggered an interruption of the gating signal. The simulated arrhythmic events (atrial fibrillation, premature atrial contraction, frequent multifocal PVC, bigeminy, trigeminy, 2–11 PVCs and VT) were identified as abnormally short heartbeats and further triggered an interruption of the gating signal. The recorded response time for all simulated scenarios and ECG lead configurations was below 5 ms. An illustrative example is shown in **Figure 5**: the gating signal is interrupted after the occurrence of the first of a pair of consecutive PVCs. The gating signal resumes after three consecutive normal heartbeats.

**Figure 5:**
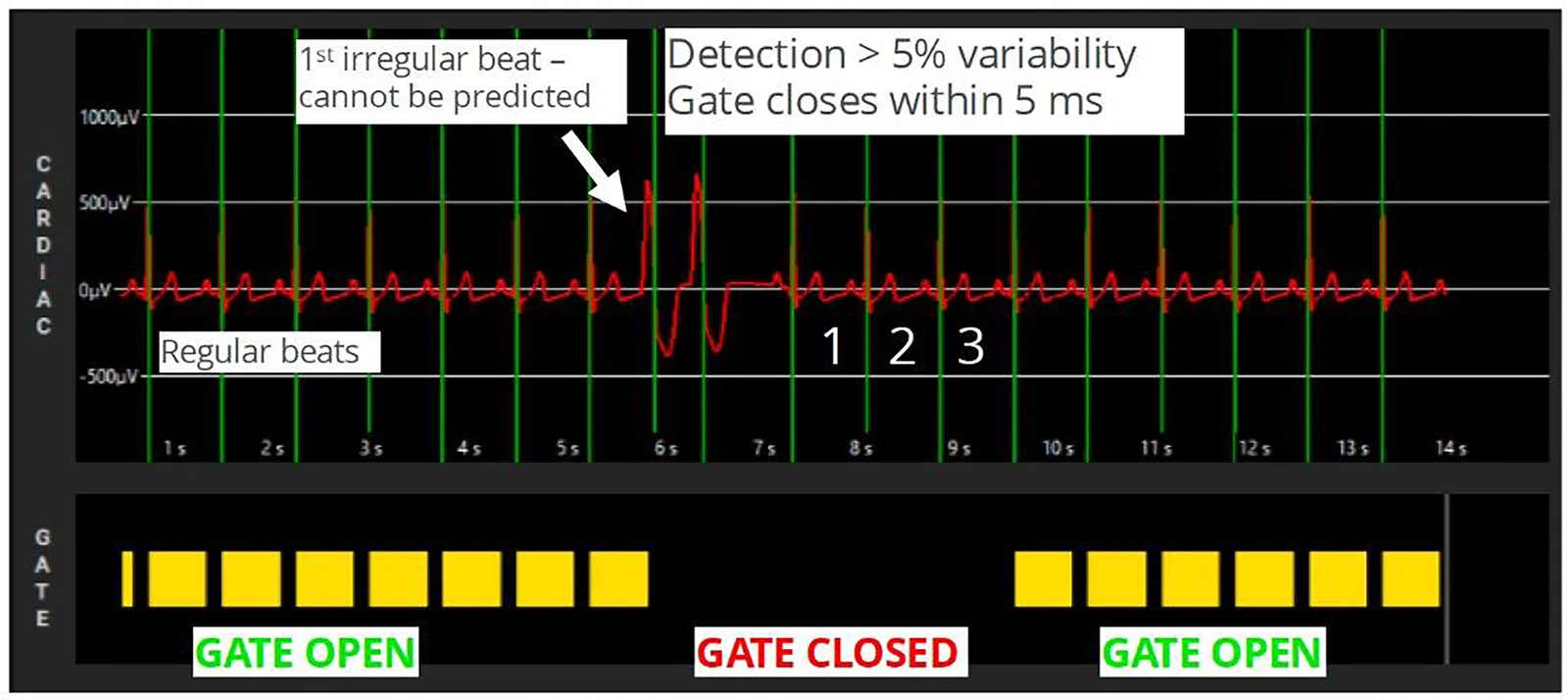
Screenshot from the CardioKit GUI showing: the ECG signal (red) and related R-peaks (green) and the timing of the gating signal (yellow).

### 3.3 Validation of gated ablation workflow

#### 3.3.1 General

The first two cases were used as tests of the protocol. They slightly differed in target size and treatment planning approach. All six subjects went through the intended workflow without major hurdles. CardioKit integrated well into the workflow, providing reliable respiratory and cardiorespiratory gating for all cases. Nominal vitals for the anesthetized swine showed a heart rate of 75 to 110 BPM, a respiratory rate of 15 to 25 breaths/min, and S_p_O_2_ (oxygen saturation) greater than 95%, with the heart rate gradually stabilizing over time (see **Figure 6**). No single event of CardioKit device failure due to radiation exposure was recorded throughout the study (or in previous tests).

**Figure 6:**
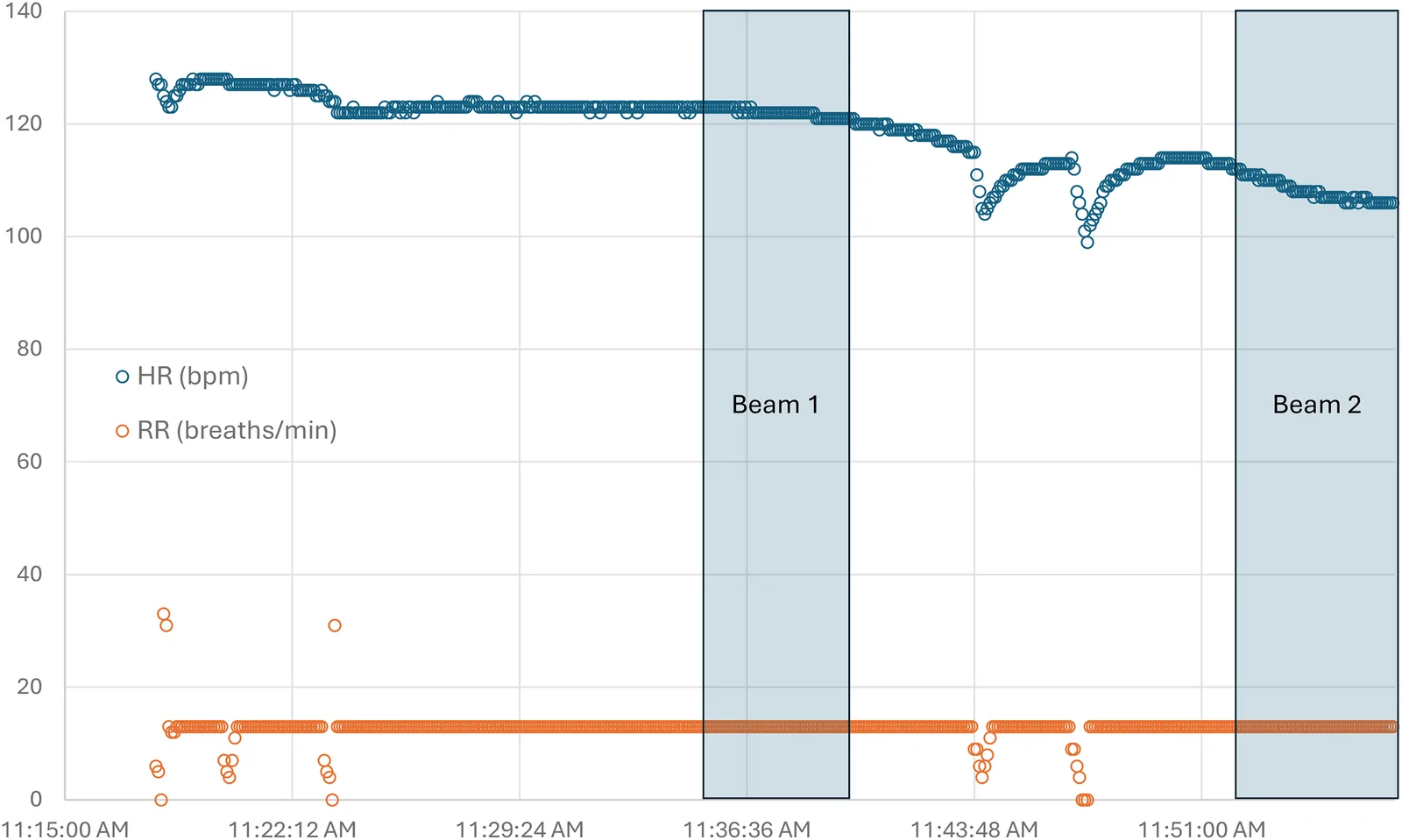
Temporal evolution of the heart rate (natural) in blue and respiratory rate (mechanical ventilation) in orange in a procedure for an example subject.

#### 3.3.2 Treatment planning

As shown in **Table 2**, treatment planning parameters differed systematically between the single-gated and dual-gated cohorts, reflecting the distinct motion management strategies employed.

**Table 2:** Summary of treatment planning parameters.

Subject case identifier	1	2	3	4	5	6
CardTV Diameter (cm)	3.5		2.5			
CardTV volume (ml)	8.95	8.78	4.8	4.81	5.02	3.41
CardTV Shape	Intersection of 3D sphere and LVFW					
CardTV Site	LVFW					
CardTV on Cardiac Phase (%)	80					
(cardiac) ITV range (%R-R)	70% to 100%	0% to 90%	70% to 100%	0% to 90%	70% to 100%	0% to 90%
(cardiac) ITV Volume (ml)	10.52	15.17	5.9	7.12	5.84	5.87
PTV margin (mm)	5					
PTV Volume (ml)	36.59	42.57	20.87	23.63	21.4	19.74
Rx ITV (Gy)	35					
Cardiac Gating	Yes	No	Yes	No	Yes	No
Cardiac Gating Window	70% to 100%	NA	70% to 100%	NA	70% to 100%	NA
Respiratory Gating	Yes	Yes	Yes	Yes	Yes	Yes
Respiratory Gating Window (mm)	2					
# of Beams	2					
Gantry angles	+10°/+300°	+270°/+345°	+265°/+320°	+270°/+350°	+280°/+350°	+270°/+330°
Repainting	No	Yes	No	Yes	No	Yes
# of Repainting	NA	4	NA	4	NA	4
CardTV to ITV expansion (%)	18%	73%	23%	48%	16%	72%
CardTV to PTV expansion (%)	409%	485%	435%	491%	426%	579%

For single-gated subjects (cases 2, 4, and 6), beam delivery spanned the full cardiac cycle (0–100% of the R–R interval), capturing the complete range of myocardial contraction and relaxation and resulting in a CardTV-to-ITV volumetric expansion of 48–73%. For dual-gated subjects (cases 1, 3, and 5), delivery was restricted to mid-to-late diastole (70–100% of the R–R interval), limiting the ITV expansion to 16–23% — a mean reduction of 45% relative to the single-gated cohort (19% ± 4% vs. 64% ± 14%). Following application of a standard 5 mm isotropic PTV margin, this difference is geometrically magnified, yielding a final PTV approximately 4.1 to 5.8 times the volume of the initial CardTV.

**Figure 7** shows the dose distribution for two arbitrarily chosen subjects (cases 3 and 4), one for each approach, cardio-respiratory and respiratory-only gating. The average DVHs based on each delivery approach are also provided. Please note that the treatment plan for case 1 was not included as it was optimized for full target coverage at the expense of the LAD (A_Coronary) dose. The impact of the chosen gating method is quite apparent given an iso-toxic treatment planning approach: ITV coverage is improved with dual vs single gating while constraining the LAD dose to a 15 GyRBE maximum.

**Figure 7:**
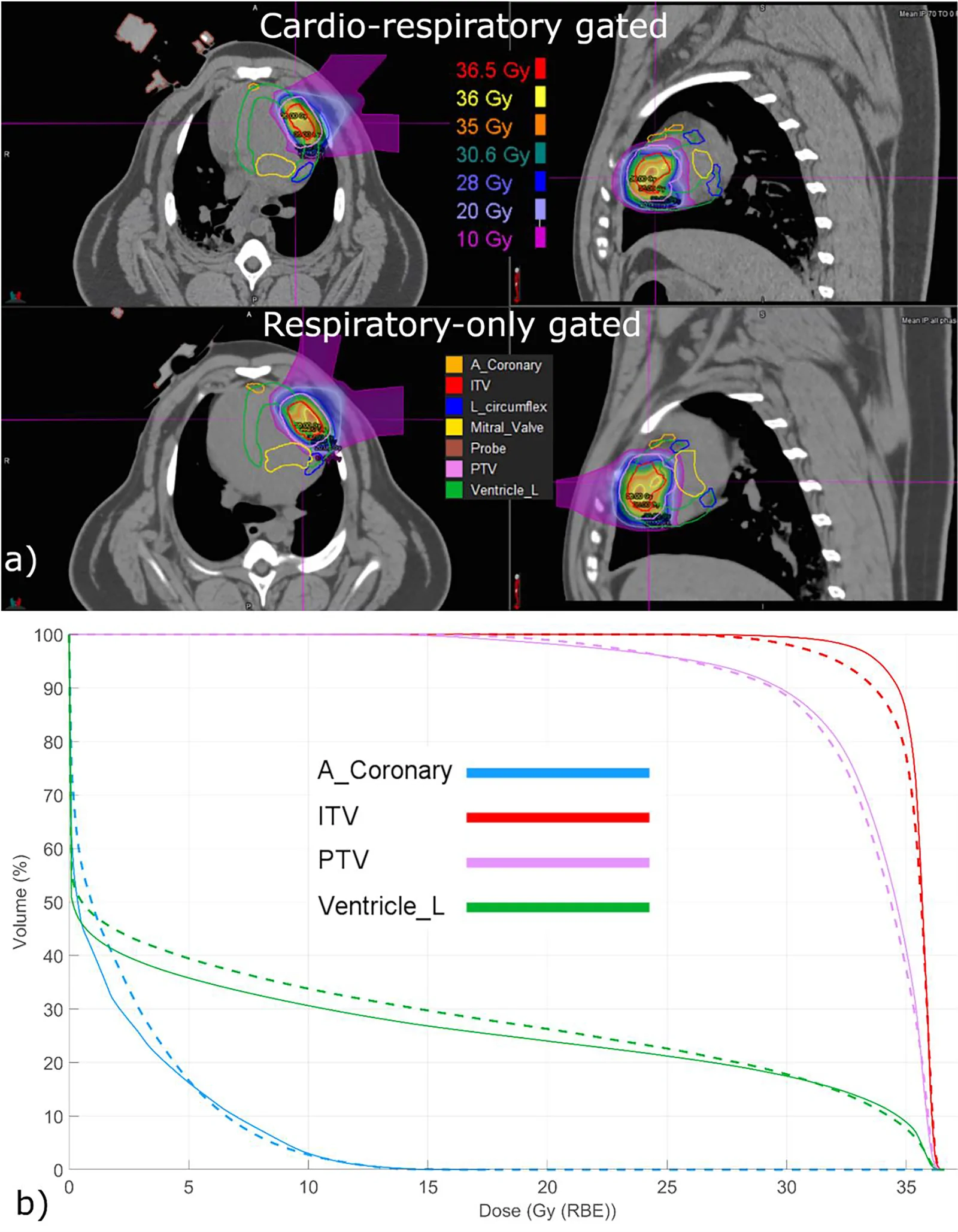
**(a)** Representative planning dose distributions for two cases, one cardio-respiratory gated and one respiratory-only gated, and **(b)** average DVHs for all cardio-respiratory-gated (solid lines) and respiratory-only-gated (dashed lines) cases – case 1 was omitted due to a difference in planning approach.

Individual dosimetric values for ITV, PTV, LAD and Ventricle are provided in the **Supplementary Table S2**.

#### 3.3.3 Beam delivery performances

The gated PBS delivery system demonstrated robust performance across all six subject irradiations, with beam delivery parameters summarized in the **Supplementary Table S1**. Each swine was treated with two gated beams. The impact of gating on total beam-on time is reflected in the delay factor, defined as the ratio of gated treatment beam-on time to non-gated Quality Assurance beam-on time. For dual-gated subjects (cases 1, 3, 5), delay factors were 8.0, 5.0, and 6.8 respectively (mean 6.6), corresponding to gated beam-on times of 1431, 677, and 1037 seconds across both beams. For single-gated subjects (cases 2, 4, 6), delay factors were substantially lower at 3.7, 2.9, and 1.5, respectively (mean 2.7), with gated beam-on times of 677, 315, and 231 seconds across both beams. The higher delay factors in the dual-gated cohort are consistent with the more restrictive combined dual duty cycle, and do not reflect any difference in dose delivery rate or ablation efficacy. Average heart rate during treatment ranged from 89 to 109 beats per minute in dual-gated subjects and 98 to 120 beats per minute in single-gated subjects, while respiratory rates ranged from 12 to 17 and 13 to 21 breaths per minute, respectively.

As shown in **Figure 8**, in the dual-gated group, 56.3% of spots (3,030 of 5,380) experienced at least one gating interruption, compared to 44.0% (2,244 of 5,099) in the single-gated group, consistent with the more restrictive combined dual duty cycle imposed by dual gating.

**Figure 8:**
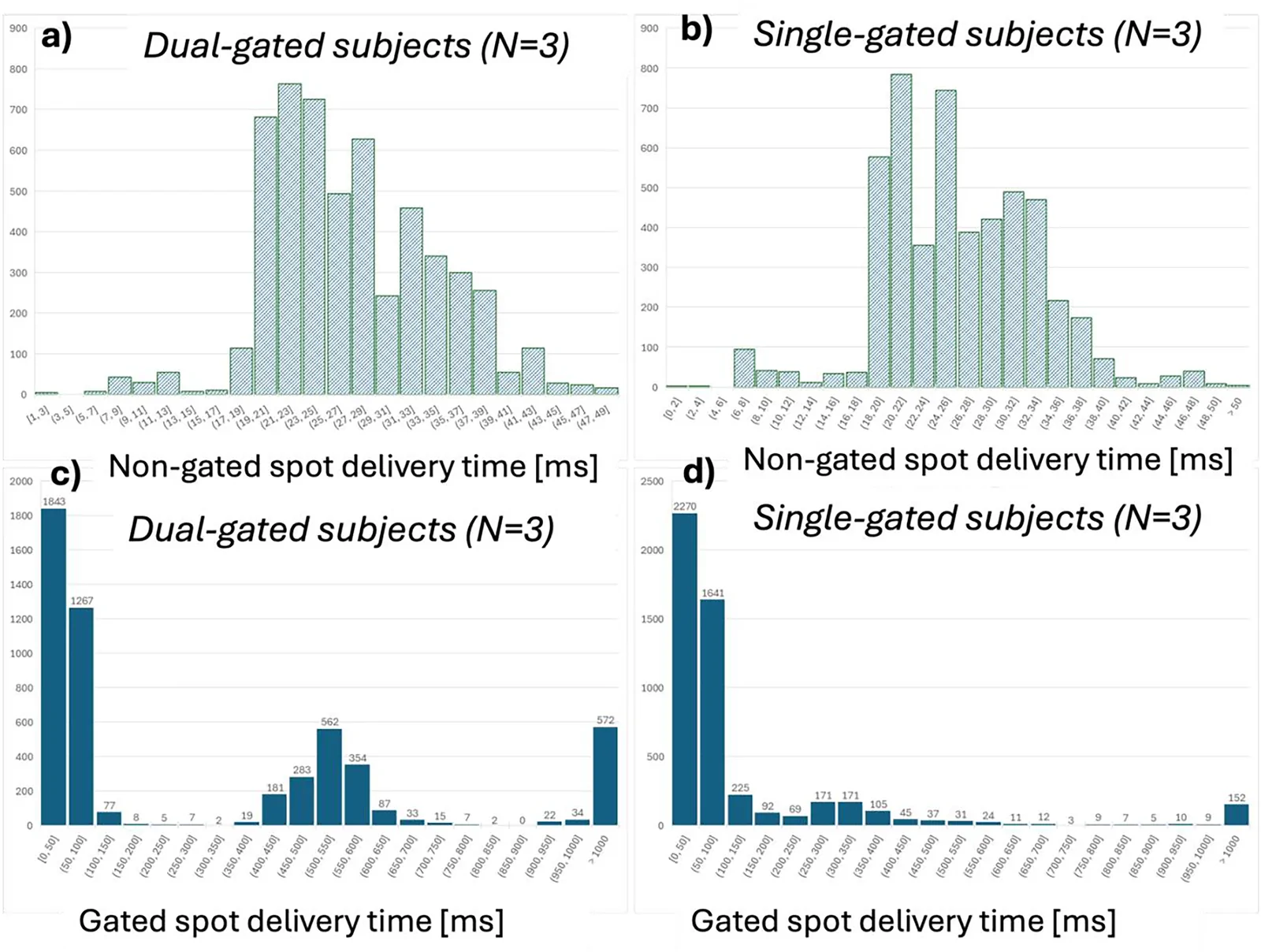
Histograms of spot delivery times from non-gated Quality Assurance deliveries of beams **(a)** for cases 1, 3, 5 and **(b)** for cases 2, 4, 6. Histograms of spot delivery times from **(c)** dual-gated treatments for cases 1, 3, 5 and **(d)** single-gated treatments for cases 2, 4, 6. For non-gated spots, the delivery times were: maximum 81/96 ms (dual-/single-gated subjects) and average 44/44 ms. For gated spots, the delivery times were: maximum 10055/3807 ms (dual-/single-gated subjects) and average 778/294 ms. Among the gated spots, 42.8% (dual-gated) and 40.8% (single-gated) experienced multiple interruptions before reaching the prescribed Monitor Units.

Among the gated spots, 42.8% and 40.8% experienced multiple interruptions before reaching the prescribed Monitor Units (MU) in the dual-gated and single-gated groups, respectively. The impact of gating interruptions on spot delivery time was substantial. For non-gated spots, average delivery times were identical between groups (44 ms), with maxima of 81 ms and 96 ms for dual- and single-gated subjects, respectively — the values are consistent with expected spot delivery durations at the cyclotron currents used in our machine, where large MU spots get split into many smaller-MU spots with a per-spot max MU limit, resulting in fast per-spot delivery. For gated spots, average delivery time increased to 778 ms in the dual-gated group and 294 ms in the single-gated group. Maximum gated spot delivery times were 10055 ms and 3807 ms, respectively, reflecting individual spots that spanned multiple gate-off intervals before accumulating their target MU. These data confirm that the mid-spot interrupt-and-resume capability of the scanning controller functioned as designed across all deliveries, with no treatment aborts due to gating timeout recorded in either cohort.

## 4 Discussion

This work demonstrates the feasibility of the upgraded PBS system and the novel workflow to image, plan and deliver proton therapy to a left ventricle target in an animal model. The successful workflow comprised the imaging of cardiac motion, contouring a target on the left ventricle, treatment planning, determination of the required cardiac gating window and finally, gated irradiation delivery. The use of cardiac gating in addition to respiratory gating was associated with 45% lower volumetric expansion of the target on average, which indicates a reduced exposure of organs at risk.

Due to the large prescription doses required for cardiac arrhythmia target ablation, the plan used relatively long beam-on times for individual pencil beam spots with respect to fast cardiac cycles. For example, with two 25 Gy Single Field Optimization beams >58% of spots would require >200 ms deliveries, which is longer than the cardiac gating window. Gating with intra-spot splitting would be essential to avoid dose spills over the outside of the gating window.

Robustness optimization was not available in the TPS at the time; as a consequence, a 5 mm PTV uniform expansion was generated for this work. This 5 mm margin corresponds to a ~4 mm setup uncertainty margin – based on repeat CT scans – and a ~2 mm margin for residual breathing motion. Robust evaluations were not explicitly performed. In a clinical context, the planning and setup protocol could be improved based on human anatomy and the available setup imaging. For instance, robustness optimization and interplay effects calculations would likely be used in lieu of a PTV.

The subjects’ average heart rate during treatment was ~102 bpm, which provided a cardiac gating window of approximately 176 ms with a 30% duty cycle (70%–100% of the R–R interval). Thus, the gate-off latency represents a small fraction of the window, minimizing unnecessary irradiation during cardiac phases outside the gating period. In the validation, system latency between the gate-off output and cessation of cyclotron output current was less than 550 µs at maximum beam current, with full ramp-up following gate reassertion taking approximately 2 ms. Both values were negligible relative to the per-spot delivery time of approximately 50 ms.

The study data was also used as test for the performance of the novel ECG and ultrasound-based CardioKit dual gating system, which provided the gating signal. Simulator-based tests of irregular heart rhythms (in particular, ectopic beats) showed that the CardioKit hardware and software were able to detect the irregularities in R-R interval and pause gating with a response time lower than 5 ms (i.e. less than 1% R-R cardiac phase at 102 bpm). This represents less than the 5% R-R cardiac phase time resolution of the cardiac 4D-CT used to model the cardiac motion at simulation.

The comparison of the CardioKit respiratory trace to a “ground-truth” respiratory 4D-CT acquired immediately after ultrasound imaging showed good agreement (1 mm difference in breathing motion amplitude and > 0.97 Pearson correlation coefficient). As expected, the ability to directly monitor the organ motion instead of using a surrogate (like for RPM) resulted in better agreement in amplitude and time (12). The possibility to image from different thorax positions, to manage the complex motion patterns of the heart (heart contraction, heart cyclical motion linked to respiration and heart drift linked to tissue relaxation) and the intrinsic robustness in terms of operator handling and image quality linked to the use of an artificial intelligence-based automatic image registration method make this technology particularly suited for STAR. Its use has been found to be feasible in terms of image quality for VT patients in supine position (43), cardiorespiratory gating of healthy pigs (23) and treatment planning for both proton (22, 44) and photon (45) treatments. Outside of animal studies, it has also been validated in phantom settings (46).

The present work can serve as a basis for the implementation of cardiorespiratory-gated STAR. For smaller target volumes in particular, the absence of cardiac gating imposes a disproportionately severe volumetric penalty, underscoring the clinical importance of cardiorespiratory gating in maximizing sparing of the surrounding healthy myocardium. All the steps described in the workflow tested in this study can be accomplished by modern radiation therapy departments. As was the case in this study, typical radiotherapy simulation scanners are not sufficiently fast for cardiac-binned 4D-CT. However, through a stoichiometric calibration, the standard scanner available at the radiology/cardiology department can be used for treatment planning. The gating window definition can be adapted based on the patient’s individual motion pattern and the isotropic margin for uncertainties in patient setup can be reduced to less than 5 mm, if more advanced imaging systems to position the patient are available. While STAR treatments-to-date have used a single fraction, baseline imaging at end of exhale was taken in the study at each session start (simulation imaging, treatment) and would be taken at each fraction start in the case of fractionated delivery.

Among important limitations of the study, the use of healthy swine and the small sample size should be emphasized. Although the use of the swine model is standard practice in the field of electrophysiology and has widespread use in the context of STAR (47), the model has shortcomings: transthoracic echocardiographic imaging in supine position is only possible for the parasternal long-axis view and the images are of lower quality than for patients. Moreover, the animal’s thorax is more curved than the relatively flatter human thorax, the heart’s respiratory motion is much smaller, and the heartbeat is significantly higher than for humans. All these aspects resulted in more difficult ultrasound probe placement and fixation and lower gating duty cycle compared to patients. Finally, the test used sedated and mechanically ventilated subjects. This enabled the comparison of non-synchronous 4D-CT, RPM and CardioKit respiratory motion curves but influenced the results in terms of duty cycle and management of respiratory rate variability. The ventilator settings were adjusted to ensure animal well-being.

## 5 Conclusion

This animal study established a feasible workflow for cardiorespiratory-gated radiation therapy of a cardiac target. Despite the limitations of the model, the workflow would be applicable to the treatment of human patients in the future. The gating controller developed to enable gated delivery was shown to perform well in terms of short latency to gating commands and effective mitigation of phantom motion. The novel CardioKit cardiorespiratory gating system used provided respiratory cardiac motion information in good agreement with state-of-the-art respiratory 4D-CT methods and integrated well into the workflow.

## Data Availability

The raw data supporting the conclusions of this article will be made available by the authors, without undue reservation.
