# Supplementary material for: Cardio-respiratory-gated proton pencil beam scanning for cardiac ablation: workflow and end-to-end validation
Source: Front Oncol. 2026 Oct 23;16:1923998. doi: 10.3389/fonc.2026.1923998 (PMC13739604; doi:10.3389/fonc.2026.1923998)
Supplement: Supplementary file 1 [file DataSheet1.pdf]

SUPPLEMENTARY MATERIALS

Table S1 Beam delivery time and delay factor

	EBAMGH_P1	EBAMGH_P2	EBAMGH_P3	EBAMGH_P4	EBAMGH_P5	EBAMGH_P6
Avg. Heart Rate (bpm)	90.8	119.8	89.3	98.2	108.8	106.0
Std	6.8	7.4	1.8	6.6	6.9	3.2
Respiratory Rate (breath per min)	11.8	12.8	14.6	20.6	16.7	14.8
Std	1.3	2.0	1.5	9.0	1.5	1.2
Cyclotron_current (nA) - Beam1	194.1	227.8	179.8	89.1	179.8	210.0
Range (cm) - Beam1	13.2	12.6	11.8	15.1	12.1	11.8
# of Energy layers - Beam1	15	15	9	6	13	11
# of Spots - Beam1	1094	1255	735	688	697	707
Cyclotron_current (nA) - Beam2	227.8	210.0	227.8	79.0	194.1	247.3
Range (cm) - Beam2	11.5	12.4	11.0	15.9	12.9	11.0
# of Energy layers - Beam2	11	13	9	7	11	10
# of Spots - Beam2	1168	1016	792	631	875	789
QA - Beam1 - Beam-on (s)	43.1	45.5	27.0	27.7	28.9	29.0
QA - Beam1 - Energy switching (s)	51.1	46.2	37.8	25.3	44.8	46.2
QA - Beam2 - Beam-on (s)	50.4	40.5	33.4	26.3	35.3	31.6
QA - Beam2 - Energy switching (s)	35.0	53.1	37.5	30.9	43.7	45.9
Tx - Beam1 - Beam-on (s)	648.1	345.2	345.2	83.8	333.0	82.7
Tx- Beam1 - Energy switching (s)	83.3	73.8	73.8	31.6	43.6	42.9
Tx - Beam2 - Beam-on (s)	652.3	197.0	197.0	74.9	453.5	68.6
Tx - Beam2 - Energy switching (s)	47.0	61.2	61.2	124.9	207.0	36.7
Beam-delivery (s) - QA without gating	179.7	185.4	135.6	110.1	152.7	152.6
Beam-delivery (s) - Tx - with gating	1430.7	677.3	677.3	315.2	1037.1	230.8
Delay factor due to gating	8.0	3.7	5.0	2.9	6.8	1.5

Table S2 Dosimetric metrics for each animal

Animal identifier	P1	P2	P3	P4	P5	P6
Gating type	Dual	Single	Dual	Single	Dual	Single
ITV D99 (GyRBE)	35.1	28.2	30.1	28.3	32.8	31.4
ITV D95 (GyRBE)	35.3	31.3	33.3	31.7	34.5	33.3
PTV D95 (GyRBE)	33.2	28.0	22.0	24.9	31.1	24.2
LAD D1 (GyRBE)	32.6	12.3	12.3	12.7	11.4	11.6
Ventricle Mean (GyRBE)	15.1	12.5	8.6	8.5	10.0	9.1

Section 1: Description of gating scanning controller logic

A novel beam-enable control logic with gating signals was implemented on the scanning controller. It holds the scanning magnet currents during beam current pauses and resumes with gating-on signals. Gating is implemented through a "Beam OK" signal architecture. An external gating source - either a respiratory motion sensor or the CardioKit system used in this study - outputs a logic-level gate signal that is routed into the PBS

control system. When the gate signal is asserted (high), the scanning controller permits beam delivery to proceed; when the gate signal is de-asserted (low), the controller withholds the beam. A custom Beam OK logic board was designed to accept and combine multiple input signals (e.g., independent respiratory gate and cardiac gate from two separate gating systems) into a single consolidated Beam OK output, delivered via fiber-optic interface. The combined logic ensures that the beam is withheld unless all active gating conditions are simultaneously satisfied: a true respiratory gate signal paired with a false cardiac gate signal will not permit beam delivery. In this study, the combined logic for cardiorespiratory gating is performed within CardioKit and only one gate signal is provided to the scanning controller. Scanning controller units operate according to a state machine that governs spot-by-spot delivery as illustrated in Figure S1.

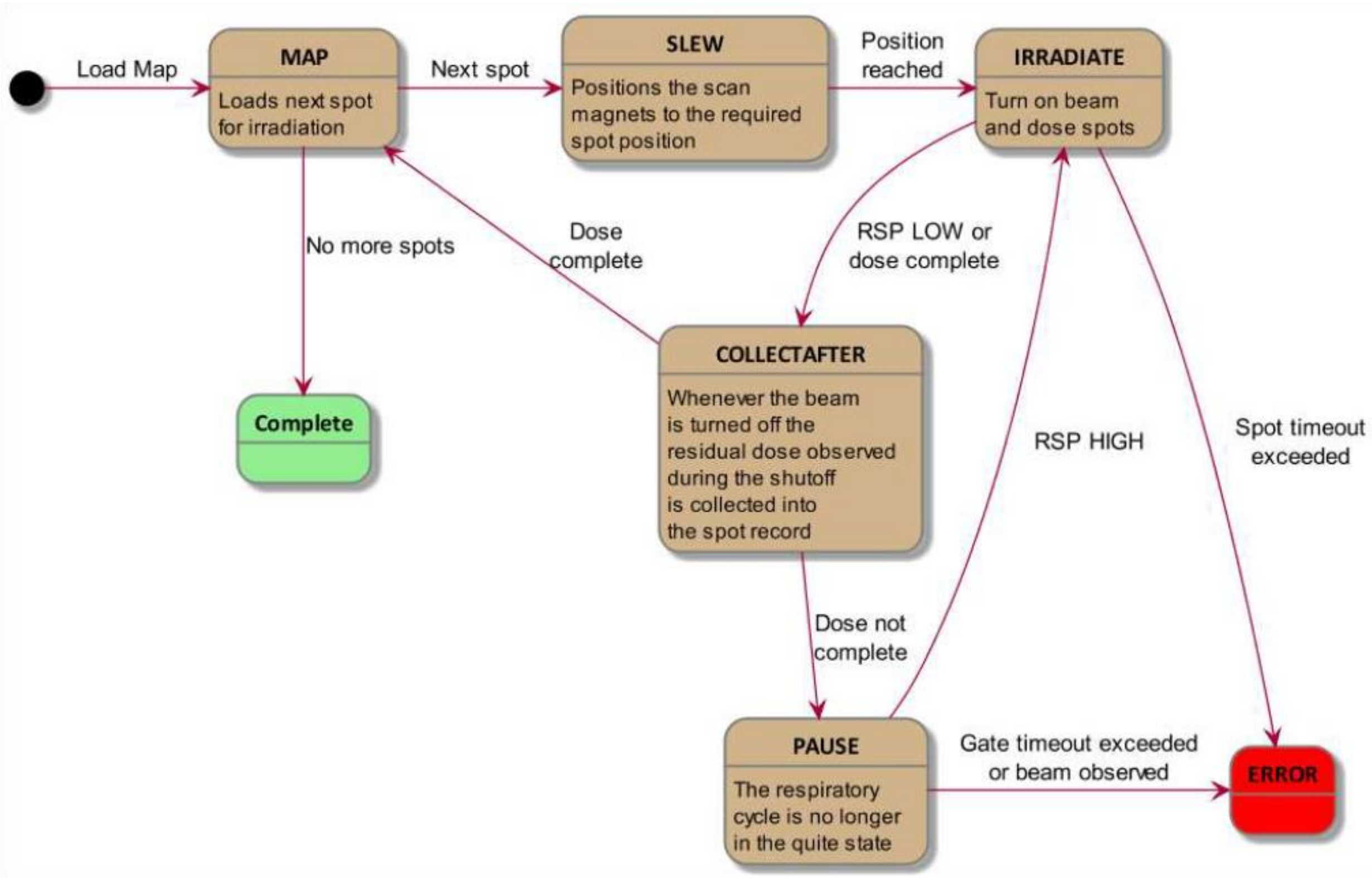

Figure S1. Scanning controller pencil beam scanning logic diagram

A total of five states relevant to gated delivery were defined: MAP (loads the next spot), SLEW (positions scanning magnets to the required spot position), IRRADIATE (turns on beam and accumulates dose), COLLECTAFTER (collects residual dose emitted during beam shut off), and PAUSE (holds while the respiratory cycle is outside the treatment window). An ERROR state is reached on any safety violation. The sequence for a gated delivery cycle is as follows:

- From the MAP state, the controller advances to SLEW, which positions the X and Y scanning magnets to the target spot coordinates. To prevent hardware damage during extended gate-off intervals, the X and Y scan amplifier currents are capped at a preset maximum (gating_max_current_x / gating_max_current_y) whenever the system is held in the PAUSE state.

- Once the magnet position is reached, the controller transitions to IRRADIATE when RSP is HIGH (beam permitted). The spot timeout counter increments only while in the IRRADIATE state; time spent in PAUSE is excluded from the timeout, and an error is generated if the timeout is exceeded during active irradiation.

- When RSP goes LOW during IRRADIATE (gate-off event), or when the spot target dose is reached, the controller transitions to COLLECTAFTER. In this state the beam is turned off, and the controller integrates any residual dose emitted during the beam shut-off transient into the spot record. This ensures no charge is lost or double-counted across gating interruptions.

- If the spot target dose was not yet reached after COLLECTAFTER, the controller enters PAUSE and holds the scanning magnet currents at the current spot position awaiting RSP HIGH. When RSP returns HIGH, the controller transitions back to IRRADIATE and delivery resumes. If RSP does not return HIGH within the configurable gating_timeout_low interval, or if beam current is detected while in PAUSE, the controller transitions to ERROR and treatment is aborted.

Section 2: Description of gating scanning controller safety checks

Various safety checks were also implemented based on a failure mode and effects analysis. Two principal hazard categories were identified: dose delivery outside the treatment window (risking healthy tissue irradiation and target underdosing), and failure to deliver dose within the window (target underdosing). Key mitigations include automatic timeout-and-abort for signal loss or a stuck-low gate; a watchdog timer and therapist monitoring for a stuck-high gate; pre-treatment polarity verification; MODE/RSP interlock logic and therapist training for mode switch errors; and scan amplifier current limits during PAUSE to prevent hardware faults. Redundancy is provided by all four scanning controllers independently receiving and acting on the Beam OK signal. The architecture was explicitly designed to permit future extension to cardiac gating, which was subsequently implemented in this study.
